# Esophageal adenocarcinoma and obesity: peritumoral adipose tissue plays a role in lymph node invasion

**DOI:** 10.18632/oncotarget.3587

**Published:** 2015-03-14

**Authors:** Elisabetta Trevellin, Marco Scarpa, Amedeo Carraro, Francesca Lunardi, Andromachi Kotsafti, Andrea Porzionato, Luca Saadeh, Matteo Cagol, Rita Alfieri, Umberto Tedeschi, Fiorella Calabrese, Carlo Castoro, Roberto Vettor

**Affiliations:** ^1^ Department of Medicine, Internal Medicine 3, Endocrine-Metabolic Laboratory, University of Padova, Padova, Italy; ^2^ Surgical Oncology Unit, Veneto Oncological Institute (IOV-IRCCS), Padova, Italy; ^3^ Department of General Surgery and Odontoiatrics, University Hospital of Verona, Verona, Italy; ^4^ Department of Cardiothoracic and Vascular Sciences, University of Padova, Padova, Italy; ^5^ Department of Molecular Medicine, Normal Anatomy Unit, University of Padova, Padova, Italy

**Keywords:** Esophageal adenocarcinoma, adipose tissue, peritumoral microenvironment, metastasis obesity

## Abstract

Obesity is associated with cancer risk in esophageal adenocarcinoma (EAC). Adipose tissue directly stimulates tumor progression independently from body mass index (BMI), but the mechanisms are not fully understood. We studied the morphological, histological and molecular characteristics of peritumoral and distal adipose tissue of 60 patients with EAC, to investigate whether depot-specific differences affect tumor behavior. We observed that increased adipocyte size (a hallmark of obesity) was directly associated with leptin expression, angiogenesis (CD31) and lymphangiogenesis (podoplanin); however, these parameters were associated with nodal metastasis only in peritumoral but not distal adipose tissue of patients. We treated OE33 cells with conditioned media (CM) collected from cultured biopsies of adipose tissue and we observed increased mRNA levels of leptin and adiponectin receptors, as well as two key regulator genes of epithelial-to-mesenchymal transition (EMT): alpha-smooth muscle actin (α-SMA) and E-cadherin. This effect was greater in cells treated with CM from peritumoral adipose tissue of patients with nodal metastasis and was partially blunted by a leptin antagonist. Therefore, peritumoral adipose tissue may exert a direct effect on the progression of EAC by secreting depot-specific paracrine factors, and leptin is a key player in this crosstalk.

## INTRODUCTION

Esophageal adenocarcinoma (EAC) is rare, but its frequency is rapidly increasing in the Western world [1]. It is one of the most fatal malignancies, with 5-year survival rates ranging from 15% to 39% [2]. Additionally, obesity has reached epidemic proportions in Western countries [3] and obese subjects presenting increased mortality and cardiovascular morbidity also have a significant risk of developing cancer [4]. Approximately 14% of cancer-related deaths in men and 20% in women are partially ascribed to obesity [5] and the relative risk for EAC occurrence is 1.52 for each 5kg/m^2^ increase in body mass index (BMI) [6]. BMI is used to clinically to determine obesity: individuals are considered overweight with BMI≥25 kg/m^2^ and obese with BMI≥30 kg/m^2^ [1]. Interestingly, a growing number of studies suggest that adipose tissue alteration/dysfunction may represent a reliable marker of obesity and visceral adipocytes hypertrophy has been associated with dyslipidemia [7], insulin resistance and inflammation [8, 9] independently of BMI values. One of the mechanisms proposed to explain the association between obesity and cancer risk is the potential action of adipokines (e.g., leptin [10, 11], adiponectin [12], insulin growth factor-1 (IGF-1) [13]) on tumor tissue. In fact, obesity is associated with an increase in adipocyte size and an altered secretion of adipokines from adipose tissue [14].

Adiponectin exerts pro-apoptotic, anti-proliferative and anti-inflammatory effects [15-17]. The expression of its receptor (AdipoR) in tumor cells is associated with a better prognosis in gastric cancer [18] and the absence of lymph node metastasis in colorectal cancer [19]. However, because adiponectin also has a pro-angiogenic activity, increased AdipoR expression has also been associated with cancer invasiveness and/or progression [20, 21]; therefore, its role still remains controversial. IGF-1 has been suggested as another potential player in the association between visceral obesity and EAC [22] and its circulating levels are increased in obesity and other metabolic complications.

Leptin acts through endocrine, autocrine and paracrine effects and exerts pro-inflammatory, mitogenic, anti-apoptotic and angiogenic functions on different cells and tissues [23, 24]. Moreover, obesity and the involvement of leptin signaling have been associated with increased lymphangiogenesis and lymph node metastasis [25, 26], as well as increased angiogenesis [27, 28], suggesting that adipose tissue may play a critical role in influencing these processes during tumor development and/or progression. Guo et al. observed increased leptin receptor (ObR) expression in several tumor tissues [29] and it has been demonstrated that leptin is able to induce epithelial-to-mesenchymal transition (EMT) in breast [30] and lung [31] cancer metastasis. In particular, the paracrine action of leptin seems to play a role in human gastric adenocarcinoma [32] and colorectal cancer [33]. Howard et al. recently found a significant correlation between upregulation of ObR and AdipoR expression in tumor tissue (but not circulating leptin levels), pathological tumor category (pT) and positive lymph node involvement of patients with EAC [34]. However, despite an abundance of epidemiological data linking obesity and cancer and a large body of *in vitro* observations confirming a relationship between leptin and obesity-related tumorigenesis, the mechanisms underlying this cross-talk remains unclear [35].

These observations suggest that peritumoral adipose tissue and adipokines may have important an role in cancer biology [36] and carcinogenesis could be induced, or at least favoured by an abundance of locally produced leptin [37]. Therefore, the aim of this study was to investigate the crosstalk between the peritumoral adipose tissue microenvironment and EAC mediated by specific adipokines, to assess a possible relationship between obesity and nodal metastasis.

## RESULTS

### Patients characteristics

A consecutive series of 60 patients who underwent esophagectomy for EAC at the Surgical Oncology Unit of the Veneto Institute of Oncology (IOV-IRCCS) were analyzed. This group consisted of patients with all adenocarcinoma histotypes and tumors were staged as shown in Table 1. 42 patients (70%) underwent neoadjuvant therapy: 5 (8.3%) had chemotherapy alone, 4 (6.6%) had radiotherapy alone and 33 (55%) had combined neoadjuvant chemoradiation. 18 patients (30%) directly underwent to esophagectomy due to their initial stage disease. Patients characteristics are outlined in Table 1.

**Table 1:** Patients characteristics

EAC patients	Normal weight (BMI < 25)	Overweight/Obese (BMI ≥ 25)
N	35	25
Gender Male:Female	4:31	3:22
Age (years)*	62 (55-72)	58 (54-65)
Tumor staging	N0 = 18 (51.4) N1 = 9 (25.7) N2 = 4 (11.4) N3 = 4 (11.4)	N0 = 16 (64) N1 = 4 (16) N2 = 2 (8) N3 = 3 (12)
Neoadjuvant therapy	26 (74.2)	16 (64)
Type of neoadjuvant therapy chemotherapy radiotherapy chemoradiotherapy	3 (8.5) 2 (5.7) 21 (60)	2 (8) 2 (8) 12 (48)

Data expressed as n(%) or

* median(IQR).

### BMI and obesity-related parameters in patients with EAC

**Figure 1:**
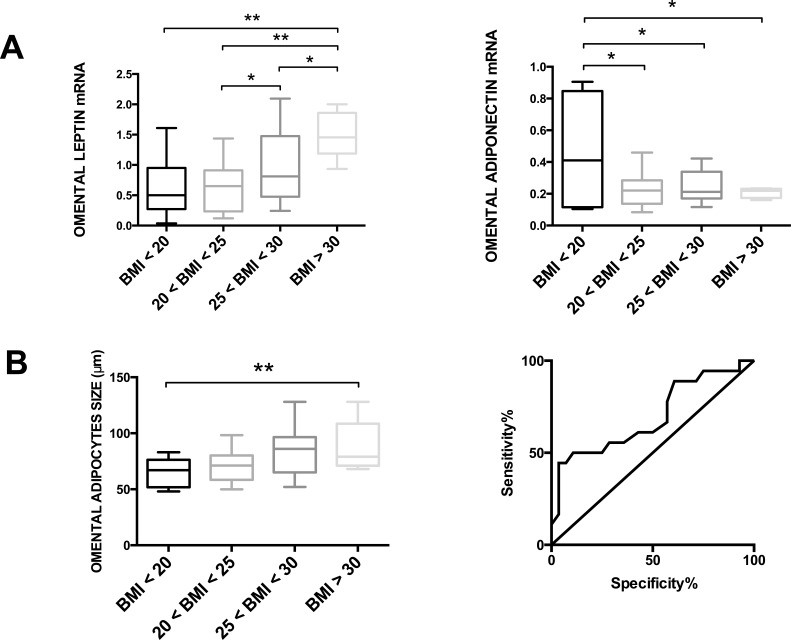
Body Mass Index (BMI) and obesity-related parameters in ADK patients At the time of surgery, a blood sample and two visceral adipose tissue biopsies (from omental and peritumoral fat depot) were collected in patients with EAC. (A) BMI values were directly correlated with leptin mRNA expression and inversely correlated to adiponectin mRNA expression of omental adipose tissue. (B) BMI values were directly correlated with adipocyte diameter in omental adipose tissue. ROC curve analysis was used to determine the best cut-off value of adipocyte diameter (= 75μm) to distinguish patients with BMI<25 (normal weight) and BMI>25 (overweight/obese). Area under curve = 0.6964; P value = 0.0259.

Anthropometric measurements, clinical data and visceral adipose tissue samples from omental and peritumoral fat depots were collected for each patient. Leptin mRNA levels in omental adipose tissue were significantly increased in patients with higher BMI, whereas adiponectin mRNA levels were decreased (Fig. 1A). We observed a direct correlation between omental adipocyte size and BMI of patients (Fig. 1B) and a ROC curve was used to determine the best cut-off value for adipocyte diameter (= 75μm), to distinguish patients with BMI<25 (normal weight) and BMI>25 (overweight/obese) (Fig. 1B). Moreover, serum levels of leptin and insulin were increased in patients with a higher BMI (Supplementary Fig. 1).

### Adipokines expression and adipocyte size in omental and peritumoral adipose tissue

**Figure 2:**
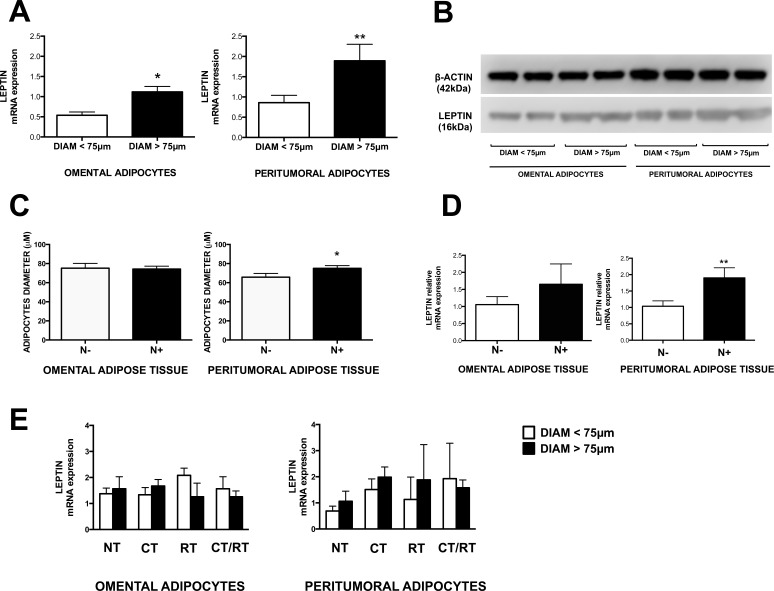
Leptin expression and adipocyte size in omental and peritumoral adipose tissue Total RNA and proteins were separately isolated from visceral adipose tissue samples of patients with EAC. Adipocytes diameters were measured in hematoxylin and eosin stained sections of the same samples. (A) Leptin mRNA expression was measured using qRT-PCR with HMBS as internal control. (B) Representative Western blot images of leptin and β-actin (used as internal control) signals in adipose tissue protein lysates. (C and D) Adipocyte size and leptin expression were measured in lymph node metastasis negative (N−) or positive (N+) EAC patients. (E) EAC patients were divided into groups based on their treatment with chemotherapy (CT), radiotherapy (RT), combined chemotherapy and radiotherapy (CT/RT) or no treatment (NT). Non-parametric statistical tests were used. *p < 0.05 and **p < 0.01.

We measured leptin, adiponectin and IGF-1 expression in adipose tissue samples from peritumoral and omental depots of patients with EAC. We observed significantly increased levels of leptin in adipocytes with diameter larger than 75μm, compared with smaller adipocytes (diameter <75μm) in both omental and peritumoral depots of patients with EAC (Fig. 2A and 2B). Adiponectin mRNA expression was slightly lower in omental adipocytes with diameter larger than 75μm, while it seemed to be rather increased in peritumoral adipose tissue with larger cells, but the observed differences were not statistically significant (Supplementary Fig. 2A). We also observed a slight but non-significant increase in IGF-1 mRNA levels in omental adipose tissue of EAC patients (Supplementary Fig. 3A).

### Lymph node metastasis and adipocyte size in omental and peritumoral adipose tissue

To explore if local tumor invasiveness could be associated with increased adipocyte size or adipokines expression in peritumoral and omental depots, we compared patients with positive nodal metastasis with patients with negative nodal metastasis, which were used as controls. We observed an increase in adipocyte diameter in peritumoral adipose tissue of patients with positive lymph node involvement (N+), compared with negative ones (N−), whereas this difference was not observed in omental adipose tissue samples (Fig. 2C). Moreover, we observed a significantly higher level of leptin mRNA expression only in peritumoral, but not omental adipose tissue of patients with nodal metastasis (N+) compared with negative controls (N−) (Fig. 2D). Adiponectin and IGF-1 mRNA levels in omental and peritumoral adipose tissue were not significantly different between N+ and N- patients (Supplementary Fig. 2B and 2C).

### Effects of neoadjuvant therapy in omental and peritumoral adipose tissue

To exclude a possible effect of neoadjuvant therapy on adipokines mRNA expression and/or adipocyte size in adipose tissue specimens, we measured these parameters in EAC patients who received chemotherapy (CT), radiotherapy (RT), both radiotherapy and chemotherapy (CT/RT) or no therapy (NT). We did not observe any significant difference in leptin mRNA expression or adipocyte diameter that could be related to the presence of neoadjuvant treatment, in either omental or peritumoral depots of patients (Fig 2E). Adiponectin and IGF-1 mRNA expression was not associated with the presence or absence of neoadjuvant therapy as well (Supplementary Fig. 2C and 3C).

### Podoplanin and CD31 expression and adipocyte size in omental and peritumoral adipose tissue

**Figure 3:**
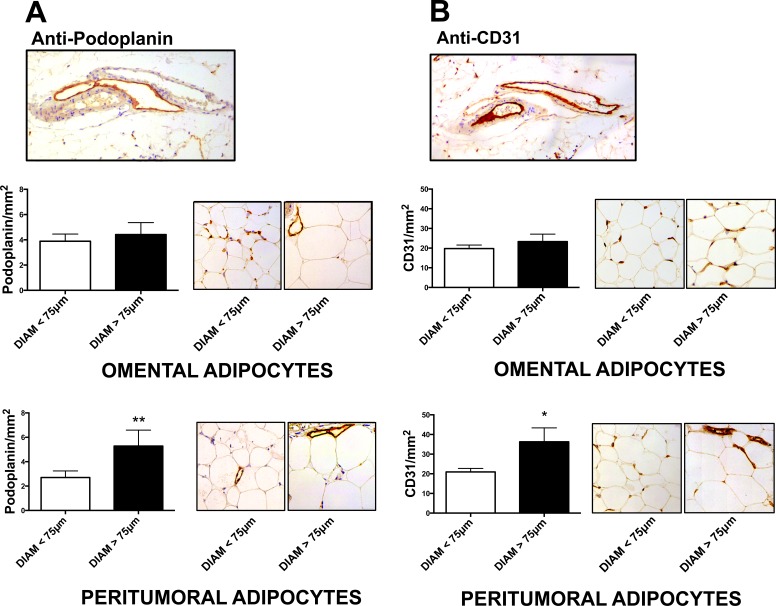
Lymphangiogenesis and angiogenesis markers in omental and peritumoral adipose tissue Adipocyte diameter and immunohistochemical expression of podoplanin (A) and CD31 (B) were evaluated in hematoxylin and eosin stained sections. Representative images of specific staining of lymphatic (A) and blood (B) vessels in two consecutive tissue sections are reported in the upper part of panel. For both markers, positive cells per mm^2^ were counted in omental and peritumoral adipose tissue depot of each patient. Non-parametric statistical tests were used. *p < 0.05 and **p < 0.01.

We measured immunohistochemical expression of Podoplanin (a marker of lymphangiogenesis) and CD31 (a marker of angiogenesis) in sections of peritumoral and omental adipose tissue of EAC patients. We observed higher levels of lymphangiogenesis (Fig. 3A) and angiogenesis (Fig. 3B) in adipose tissue samples with adipocytes diameters larger than 75μm, compared to those with smaller adipose cells (diameter <75μm). This difference, although observed in both depots, was statistically significant only in peritumoral and not omental adipose tissue. Moreover, podoplanin positive staining in peritumoral (but not omental) adipose tissue was associated with nodal metastasis in EAC patients (R= 0.29, p=0.02).

### Effects of adipose tissue conditioned medium (CM) on leptin and adiponectin receptors in OE33 cells

**Figure 4:**
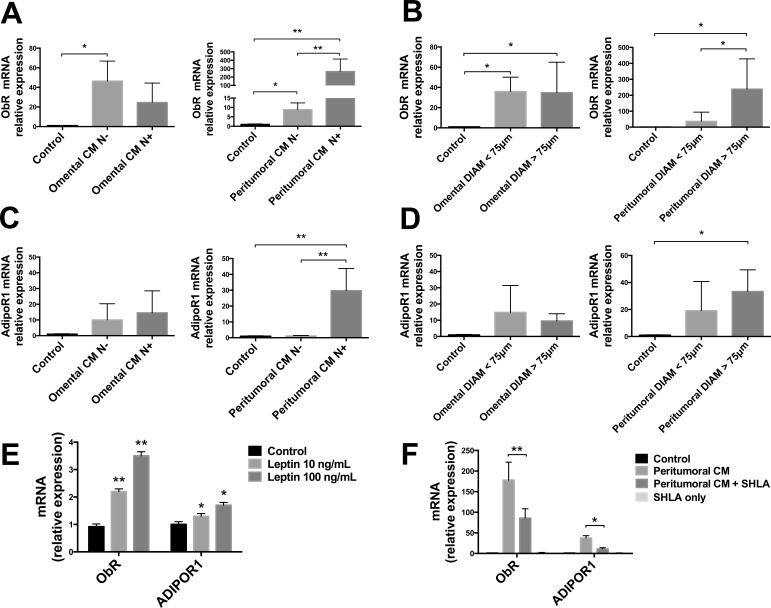
ObR and AdipoR1 mRNA expression analysis in OE33 cells Human EAC cells (OE33) were cultured with conditioned medium (CM) derived from adipose tissue fragments of omental and peritumoral depots of EAC patients. After 48 h, mRNA levels of ObR and AdipoR1 were measured using qRT-PCR with 18s rRNA and HMBS as internal controls. (A and C) Lymph node negative (N−) or positive (N+) involvement was estimated by TNM values for each patient. (B and D) Adipocyte diameters were measured in hematoxylin and eosin stained sections of the same samples. OE33 cells were cultured for 48 h in the presence of human recombinant leptin (E) or in the presence of leptin antagonist SHLA in addition to peritumoral CM treatment (F). *p < 0.05 and **p < 0.01.

To further investigate the potential mechanism by which adipose tissue could influence tumor physiology, we analyzed the effects of a conditioned medium (CM) derived from adipose tissue cultures on an EAC cell line (OE33 cells). We measured the mRNA expression of the leptin receptor (ObR) and the adiponectin receptor 1 (AdipoR1) in OE33 cells after a 48 h treatment with medium collected from adipose tissue culture of peritumoral and omental biopsies of patients. We observed that mRNA expression of ObR and AdipoR1 was generally increased in cells treated with CM of both peritumoral and omental adipose tissues, compared with untreated control cells (Fig. 4). In particular, we observed a dramatic increase in ObR mRNA expression in cells treated with CM derived from patients with lymph node involvement (N+) compared with negative patients (N−) in peritumoral, but not omental adipose tissue (Fig. 4A). We also observed a higher ObR mRNA expression in cells treated with CM derived from peritumoral adipose tissue with larger adipocytes (diameter >75μm) compared with CM derived from tissue with smaller adipocytes, but this difference was not observed in cells treated with omental adipose tissue CM (Fig. 4B). AdipoR1 mRNA expression was massively increased in cells treated with CM of N+ patients only in peritumoral, but not omental adipose tissue (Fig. 4C) and a slightly increased expression was also observed in peritumoral adipose tissue with cells larger than 75μm (Fig. 4D).

To confirm our hypothesis of a potential action of leptin in altering mRNA expression in OE33 cells, we performed a 48 h treatment with two different concentrations of human recombinant leptin (10 ng/mL and 100 ng/mL). We observed a dose-dependent increase in ObR and AdipoR1 mRNA expression in cells treated with leptin compared with untreated control cells (Fig. 4E). To further confirm the involvement of leptin signalling we treated the cells with a leptin antagonist (SHLA) and found that the presence of SHLA partially blunted the increasing effect of peritumoral adipose tissue CM on ObR and AdipoR1 mRNA expression in OE33 cells (Fig. 4F).

### Effects of adipose tissue conditioned medium (CM) on α-SMA and E-cadherin expression in OE33 cells

**Figure 5:**
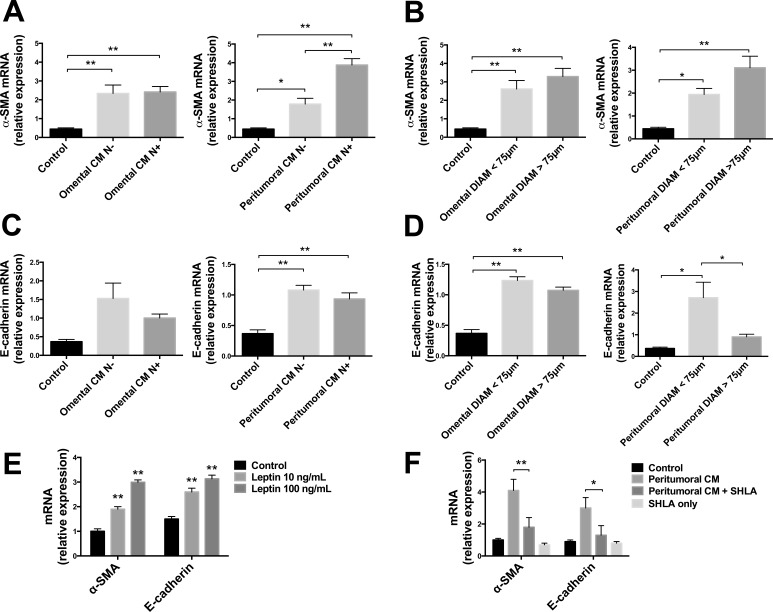
α-SMA and E-cadherin mRNA expression analysis in OE33 cells Human EAC cells (OE33) were cultured with conditioned medium (CM) derived from adipose tissue fragments of omental and peritumoral depots of EAC patients. After 48h, mRNA levels of α-SMA and E-cadherin were measured using qRT-PCR with 18s rRNA and HMBS as internal controls. (A and C) Lymph node negative (N−) or positive (N+) involvement was estimated by TNM values for each patient. (B and D) Adipocyte diameters were measured in hematoxylin and eosin stained sections of the same samples. OE33 cells were cultured for 48 h in the presence of human recombinant leptin (E) or in the presence of leptin antagonist SHLA in addition to peritumoral CM treatment (F). *p < 0.05 and **p < 0.01.

To explore if the treatment with CM could possibly affect the EMT process in OE33 cells, we measured the mRNA expression of alpha-smooth muscle actin (α-SMA) and E-cadherin (molecular markers of motility and adhesion, respectively) after a 48 h treatment. We observed a significant increase in α-SMA and E-cadherin mRNA expression in OE33 cells treated with both omental and peritumoral CM, compared with untreated control cells (Fig. 5). Interestingly, the mRNA expression of α-SMA was significantly higher in cells treated with CM derived from patients with lymph node involvement (N+) compared with negative patients (N−), but this difference was not found in cells treated with omental CM (Fig. 5A). We also observed an increase of α-SMA mRNA expression in cells treated with CM derived from both adipose tissue with larger adipocytes (diameter >75μm) and this difference was more evident in peritumoral depot (Fig. 5B). The mRNA expression of E-cadherin tended to be decreased in cells treated with CM derived from N+ patients compared to those treated with CM of N- patients (Fig. 5C), whereas we observed a significant down-regulation of E-cadherin expression in cells treated with CM derived from peritumoral adipose tissue with larger adipocytes, compared with CM derived from samples with smaller cells (Fig. 5D). α-SMA and E-cadherin mRNA levels were increased in a dose-dependent manner in OE33 cells treated with human recombinant leptin (Fig. 5E) and the treatment with SHLA in the presence of peritumoral adipose tissue derived CM showed a significant reduction of its increasing effect on α-SMA and E-cadherin mRNA expression (Fig. 5F).

## DISCUSSION

This study began from the observation that despite obesity being a well established risk factor for several types of cancer, the correlations between “classic” obesity parameters (e.g.: BMI, circulating adipokines levels, hyperlipidemia, insulin resistance) and tumor staging/progression are not always consistent, suggesting that the influence of obesity on cancer behaviour should be investigated more thoroughly, especially in the context of depot-specific adipose tissue pathophysiology. Moreover, a significant correlation between upregulation of ObR and AdipoR expression in tumor tissue and nodal metastasis in patients with EAC was recently observed [34]. Therefore, we measured different anthropometric, physiological, histochemical, tumoral and molecular parameters in 60 patients affected by EAC, to investigate the potential interplay between peritumoral adipose tissue and cancer in terms of local invasiveness.

Our results showed a clear association between leptin mRNA expression and adipocyte size (a marker of obesity) in patients with EAC. Adipocyte size was also associated with higher expression of lymphangiogenesis and angiogenesis markers. These findings are in line with previous observations [38], however we indentified for the first time a stronger correlation between adipocytessize and obesity-related alterations in the periesophageal depot, compared with the distal one (omental) of EAC patients. Interestingly, the increased adipocyte size and the higher levels of leptin mRNA expression were strongly associated with positive nodal metastasis only in peritumoral but not omental adipose tissue of patients, suggesting that contiguous visceral adipose tissue may directly affect tumor invasiveness. This relationship may not be detected by measuring the traditional systemic obesity-related parameters. In fact, BMI as well as omental depot features did not show any direct relation with EAC nodal status, indicating that fat mass distribution is not univocal, and different visceral fat depots can influence different pathophysiological processes, as has already been observed for cardiovascular complications [39, 40]. All of these results highlight the role of peritumoral adipose tissue in the interplay between obesity and cancer progression.

**Figure 6:**
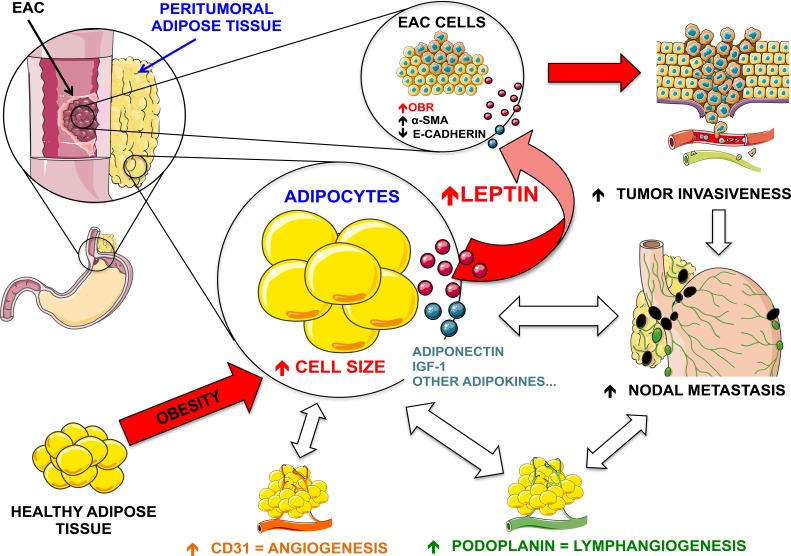
Possible mechanism of crosstalk between peritumoral adipose tissue and tumor cells in EAC Obesity is associated with increased adipocyte size and altered secretion of several adipokines from adipose tissue. Peritumoral adipose tissue with increased cell diameter expresses higher levels of leptin, an adipokine that exerts a paracrine action on EAC cells by up-regulating the expression of EMT markers such as α-SMA and E-cadherin, and thus likely promotes the invasiveness of tumor. Moreover, increased adipocyte diameter in the peritumoral depot is also associated with increased levels of CD31 (an angiogenesis marker) and podoplanin (a lymphangiogenesis marker) and this enrichment of blood vessels and lymphatic vessels may represent a permissive condition that further supports the progression/invasion of tumor cells. In fact, leptin and podoplanin expression in peritumoral (but not distal) adipose tissue is strongly associated with nodal metastasis (N) in EAC patients. Figure created using Servier Medical Art (http://www.servier.com/Powerpoint-image-bank) by Servier, under CC BY 3.0.

To explore whether chemotherapy and/or radiotherapy could have influenced our data by modifying the molecular processes of peritumoral or distal adipose tissue, we investigated the effect of neoadjuvant treatment on the parameters that we analyzed in EAC patients. It has been shown that tumor leptin mRNA expression may be associated with chemo-resistance; however it is also correlated to a therapy-independent better prognosis [41]. Thus we measured leptin, adiponectin and IGF-1 mRNA expression, as well as adipocyte diameter in both omental and peritumoral adipose tissue of patients treated with chemotherapy, radiotherapy, chemo- and radiotherapy or no treated at all. We did not observe any significant influence of neoadjuvant therapy in the parameters that we analyzed, confirming that the results are not impaired by a cytotoxic effect. Most patients stopped their preoperative chemo-radiation at least 8 weeks prior to surgery, so the impact of neoadjuvant therapy on peritumoral adipose depot may be progressively faded.

To better clarify the involvement of the peritumoral adipose tissue in the direct crosstalk with esophageal cells, we cultured a human EAC cell line (OE33) in the presence of conditioned media derived from isolated adipose tissue explants (peritumoral and omental) of patients with EAC. We observed a substantial response in terms of ObR and AdipoR1 mRNA upregulation in the esophageal cells cultured with adipose tissue conditioned media, compared with control cells maintained in standard medium. These results suggest that the leptin and adiponectin signaling pathways are involved in the paracrine action of adipose tissue in EAC. Interestingly, we observed a dramatic increase in ObR and AdipoR1 mRNA expression in OE33 cells cultured with medium derived from peritumoral adipose tissue of patients with positive lymph node involvement (N+) compared with medium derived from adipose tissue of lymph node negative patients (N−). Moreover, ObR expression was increased in cells treated with CM derived from peritumoral adipose tissue containing larger cells. These results suggest that the peritumoral adipose tissue can play a direct role in the relationship between obesity and EAC, potentially influencing local cancer invasiveness. One mechanism underlying this relationship may depend on the local secretion of adipokines, specifically leptin, and their paracrine effect on tumor behavior.

We also observed that OE33 cells cultured with CM derived from adipose tissue of EAC patients had a significant increase in α-SMA and E-cadherin mRNA expression compared with untreated cells, suggesting that in EAC cells, the molecular processes of migration and adhesion are influenced by factors secreted from adipose tissue. In particular, the increase in α-SMA mRNA expression was higher in cells treated with CM derived from peritumoral adipose tissue of patients with positive lymph node involvement (N+) than in those treated with the CM from lymph node negative ones. The increase of E-cadherin mRNA expression was significantly lower in cells treated with CM derived from peritumoral adipose tissue with larger cells, in comparison with those treated with CM derived from tissue with smaller cells. These results showed that the peritumoral adipose tissue may also influence EMT processes and the hypertrophy of adipose cells may have a direct effect on cancer progression. It has been previously shown that impaired adipose tissue-derived secretory products can promote mobilization and EMT processes [42]; however our study analyzed this phenomenon in EAC cells and we highlight for the first time the specific role of peritumoral adipose tissue in exerting a paracrine action on tumor cells biology that clearly differs from the distal visceral depot.

We finally observed that a 48 h treatment of OE33 cells with recombinant leptin promotes the same changes as CM derived from adipose tissue of EAC patients in terms of ObR, AdipoR1, α-SMA and E-cadherin mRNA levels; however, the differences in terms of relative expression units are noticeably lower than those induced by CM treatment. Moreover, the administration of a leptin antagonist during the treatment with peritumoral adipose tissue-derived CM partially reduced its effect on ObR, AdipoR1, α-SMA and E-cadherin mRNA expression, but did not completely blunted their levels as those of untreated cells. This observation suggests that adipose tissue, in particular the peritumoral tissue, may locally secrete a variety of cytokines, in which leptin plays a crucial role. In fact, its specific inhibition greatly decrease the peritumoral adipose tissue effect on EAC cells gene expression, suggesting that several factors could influence tumor behavior via a synergistic action that is partially driven by leptin.

Furthermore, this study suggests that the prevention or inhibition of potentially negative effects of adipose tissue on cancer progression should be considered in the therapeutic approach of EAC patients. It was shown that obesity, leptin levels and adipocyte size can be reduced by rapamycin, an immunosuppressive chemical able to bind mTOR Complex 1 (mTORC1) and inhibit downstream signaling [43, 44]. More importantly, rapamycin might prevent cancer onset possibly in part due to these effects on fat tissues [45]. Therefore, a treatment with well-known conventional compounds that are able to reduce the metabolic alterations induced by obesity, such as rapamycin, may warrant further testing to be introduced into the clinical management of EAC.

In conclusion, our results clearly show that the crosstalk between adipose tissue and tumor cells is deeply influenced by depot-specific differences in EAC patients. Moreover, peritumoral adipose tissue with increased cell size (a hallmark of obesity) may be a permissive milieu for local tumor invasiveness, and we showed that an increased secretion of leptin is strongly associated with alterations in the mRNA levels of key regulator genes of tumor progression. All of these considerations are particularly important for the clinical evaluation of patients with cancers affecting the digestive system, where the main traits of obesity are often “lost” after severe cachexia and rapid weight loss.

## MATERIALS AND METHODS

### Study design

Adipose tissue and blood samples from 60 consecutive patients with EAC who underwent esophagectomy at the Surgical Oncology Unit of the Veneto Institute of Oncology, were prospectively collected and analyzed. Complete anthropometric and tumor staging data have been retrieved for each patient. The study was performed in accordance with the principles of the Helsinki Declaration and all potential patients were asked to give written consent to have their data collected. The study obtained the approval of the Ethical Committee of Veneto Institute of Oncology.

### Preoperative staging

Tumor–node–metastasis (TNM) staging was performed according to the criteria of the International Union Against Cancer [46]. Based on preoperative staging and according to the recommendations of the multidisciplinary team work-up, patients with tumors staged above T3N0 or any T N1 were considered suitable for neoadjuvant therapy. Patients were considered resectable when staged below T3N0 or, after the termination of neoadjuvant treatment, when there was no evidence of distant metastases or locally advanced tumors with gross periesophageal involvement at restaging.

### Neoadjuvant therapy

The most common preoperative chemotherapy regimen for patients who were prescribed combined modality therapy consisted of 5-fluorouracil (5-FU) and a platinum agent (standard regimen for treatment was DDP 100 mg/m2 on day 1, and 5-FU 1000 mg/m2 per day in continuous infusion from day 1 to day 5 for 3-4 cycles). Chemotherapy was usually administered concurrently with radiation therapy, but the exact sequence depended on either the clinical protocol or on the single physician's preference.

Radiation was usually administered in daily fractions of 1.8 Gy for a total dose of 45 to 50 Gy. For carcinoma of the lower third esophagus, the field was extended to include both the perigastric and celiac nodes. This involved an initial phase using anteroposterior/posteroanterior fields with a total dose up to 30.6 Gy in 1.8 fractions. The radiation portals were then modified to encompass the primary tumor and metastatic nodes with 2 cm margins using a oV-cord conformal oblique Welds with a total dose up to 45 Gy.

Patients prescribed only chemotherapy underwent two cycles of cisplatin in combination with 5-FU before surgery. After the second cycle of chemotherapy, the tumor was restaged and patients with stable or progressive disease underwent surgery without further delay. A third cycle of chemotherapy was initiated in those patients who responded to the first two cycles. In all cases, surgery was performed 3-4 weeks after the last cycle of chemotherapy [47].

### Surgical resection, adipose tissue and blood sample collection

Details concerning surgical techniques have been published elsewhere [48]. Briefly, esophagectomy was performed using an Ivor Lewis procedure, via a laparotomy or laparoscopy and right thoracotomy, for tumors of the mid lower esophagus and the gastric cardia. At least 6–8 cm of healthy esophagus was resected above the proximal edge of the tumor to avoid neoplastic involvement of the resection margins. In this group of patients, an en bloc lymph node dissection was performed. The alimentary tract was reconstructed using the gastric pull-up technique; if the stomach was unavailable, either a jejunal loop or the left colon was used. Patients were seen during the follow-up period by the operating surgeons at regularly scheduled intervals (after 1, 3, 6, 12 months and every 6–12 months, thereafter).

During surgical procedures, two biopsies of visceral adipose tissue were collected from each patient, one from periesophageal depot (2 cm close to cardia) and one from omental depot. A sample of whole blood was obtained concurrently. Fresh specimens were rapidly divided and prepared for *in vitro* culture, or immediately frozen in liquid nitrogen, or fixed in formalin for subsequent analysis. Blood samples were centrifuged for 10 min at 3500 rpm at 4°C, to separate serum.

### Adipose tissue conditioned medium and OE33 cell treatment

Fresh adipose tissue samples were immediately washed in sterile PBS at 37°C, separated from major vessels and fibers, minced and transferred in a sterile multi-well plate containing RPMI 1640 medium (Sigma) supplemented with 10% fetal bovine serum and 2mM glutamine. Samples were maintained at 37°C in a sterile humidified atmosphere of 5% CO_2_ and after 48 h conditioned medium was collected.

The EAC OE33 cell line was purchased from Sigma and maintained in RPMI 1640 medium (Sigma) supplemented with 10% foetal bovine serum, 150U/mL streptomycin, 200U/mL penicillin and 2mM glutamine (Life Technologies). At 60% confluence, cells were washed twice in warm sterile PBS and medium was replaced by simple culture medium (control cells) or conditioned media previously collected from cultured adipose tissue biopsies (CM treated cells). Leptin and leptin antagonist treatments was performed by adding 10 ng/mL or 100 ng/mL recombinant human leptin (R&D Systems) or 0.7 ng/mL Super human leptin antagonist (SHLA, Protein Laboratories Rehovot Ltd) to cells. After 48 h of treatment, cells were washed twice with warm PBS and harvested for gene expression analysis.

### RNA isolation and real-time quantitative PCR

Total RNA was isolated from freshly frozen visceral adipose tissues and OE33 cells using the Rneasy Lipid Tissue Mini Kit and the Rneasy Mini Kit (Qiagen), respectively, treated with DNase (TURBO-DNase-free, Life Technologies) and reverse transcribed using random primers (Promega) and M-MLV reverse transcriptase (Promega). mRNA levels were measured by Real-Time PCR (DNA Engine Opticon2, MJ Research) using SYBR Green PCR Master Mix (Life Technologies) and specific intron-spanning human primers, according to manufacturer's instructions. Values were calculated as the mean of triplicate measurements and levels were normalized to HMBS mRNA expression.

### Protein isolation and western blot analysis

Proteins were isolated from visceral adipose tissues using T-PER Mammalian Protein Extraction Reagent (Thermo Scientific) as indicated by the manufacturer, in the presence of a cocktail of protease and phosphatase inhibitors (Thermo Scientific). Protein content was determined by the bicinchoninic acid protein assay (Thermo Scientific) and 70 μg of proteins were separated with SDS-PAGE under reducing conditions. The separated proteins were then electrophoretically transferred to a nitrocellulose membrane (BioRad). Proteins of interest were revealed with specific antibodies: Ob Antibody (H-146) sc-9014 (Santa Cruz) and anti-β actin (Sigma-Aldrich). The immunostaining was detected using horseradish peroxidase-conjugated anti-rabbit or anti-mouse immunoglobulin (Novex), bands were revealed by the SuperSignal Substrate (Pierce), detected using the C-Digit Blot Scanner and quantified by densitometry using the Image Studio software (all from Li-Cor).

### Immunohistochemistry

Formalin-fixed adipose tissue samples were paraffin-embedded and adipocytes diameter and distribution were evaluated by using morphometrical approach in hematoxylin and eosin stained sections (4 μm). Consecutive serial sections were then immunostained for Podoplanin (Anti-D240) and CD31 (Anti-CD31) (all from AbCam) and counterstained with hematoxylin. Ten areas (1 mm^2^) for each section were analyzed and all the parameters were measured by computer-assisted morphometric analysis (Image Pro-plus version 5).

### Serum analysis

Serum samples of patients were briefly centrifuged at 1000g at 4°C and cytokines levels were analyzed by multiplex ELISA using an Adipokine Human Panel B Milliplex assay (Millipore). Circulating concentrations of leptin and insulin were measured in duplicate for each patient following manufacturer's instructions.

### Statistical analysis

All statistical analyses were performed using GraphPad Prism software version 5.0 (GraphPad Software, San Diego, CA). Data are shown as median and interquartile range (IQR) or frequency. Non-parametric correlations were measured using the Spearman rank correlation coefficient. The Mann-Whitney U test for two independent samples was performed to assess differences between continuous variables with Bonferroni correction for multiple comparisons when appropriated. The *in vitro* results are expressed as mean ± SEM and experiments were performed in triplicates. All the tests were two-sided and a p-value below 0.05 was considered significant.

## SUPPLEMENTARY MATERIAL, FIGURES AND TABLE



## References

[1] Jemal A, Murray T, Samuels A, Ghafoor A, Ward E, Thun MJ (2003). Cancer statistics, 2003. CA Cancer J Clin.

[2] Refaely Y, and Krasna MJ (2002). Multimodality therapy for esophageal cancer. Surg Clin North Am.

[3] Hedley AA, Ogden CL, Johnson CL, Carroll MD, Curtin LR, Flegal KM (2004). Prevalence of overweight and obesity among US children, adolescents, and adults, 1999-2002. JAMA.

[4] Wolk A, Gridley G, Svensson M, Nyren O, McLaughlin JK, Fraumeni JF, Adam HO (2001). A prospective study of obesity and cancer risk (Sweden). Cancer Causes Control.

[5] Calle EE, Rodriguez C, Walker-Thurmond K, Thun MJ (2003). Overweight, obesity, and mortality from cancer in a prospectively studied cohort of U.S. adults. N Engl J Med.

[6] Renehan AG, Tyson M, Egger M, Heller RF, Zwahlen M (2008). Body-mass index and incidence of cancer: a systematic review and meta-analysis of prospective observational studies. Lancet.

[7] Hoffstedt J, Arner E, Wahrenberg H, Andersson DP, Qvisth V, Lofgren P, Ryden M, Thorne A, Wiren M, Palmer M, Thorell A, Toft E, Arner P (2010). Regional impact of adipose tissue morphology on the metabolic profile in morbid obesity. Diabetologia.

[8] Liu A, McLaughlin T, Liu T, Sherman A, Yee G, Abbasi F, Lamendola C, Morton J, Cushman SW, Reaven GM, Tsao PS (2009). Differential intra-abdominal adipose tissue profiling in obese, insulin-resistant women. Obes Surg.

[9] Kim SH, Reaven G (2010). Obesity and insulin resistance: an ongoing saga. Diabetes.

[10] Prieto-Hontoria PL, Perez-Matute P, Fernandez-Galilea M, Bustos M, Martinez JA, Moreno-Aliaga MJ (2011). Role of obesity-associated dysfunctional adipose tissue in cancer: a molecular nutrition approach. Biochim Biophys Acta.

[11] McTiernan A (2005). Obesity and cancer: the risks, science, and potential management strategies. Oncology (Williston Park).

[12] Dalamaga M, Diakopoulos KN, Mantzoros CS (2012). The role of adiponectin in cancer: a review of current evidence. Endocr Rev.

[13] Toren P, Mora BC, Venkateswaran V (2013). Diet, obesity, and cancer progression: are adipocytes the link?. Lipid Insights.

[14] Lundgren M, Svensson M, Lindmark S, Renstrom F, Ruge T, Eriksson JW (2007). Fat cell enlargement is an independent marker of insulin resistance and ‘hyperleptinaemia’. Diabetologia.

[15] Li FY, Cheng KK, Lam KS, Vanhoutte PM, Xu A (2011). Cross-talk between adipose tissue and vasculature: role of adiponectin. Acta Physiol (Oxf).

[16] Ouchi N, and Walsh K (2007). Adiponectin as an anti-inflammatory factor. Clin Chim Acta.

[17] Goldstein BJ, and Scalia R (2004). Adiponectin: A novel adipokine linking adipocytes and vascular function. J Clin Endocrinol Metab.

[18] Tsukada T, Fushida S, Harada S, Terai S, Yagi Y, Kinoshita J, Oyama K, Tajima H, Fujita H, Ninomiya I, Fujimura T, Ohta T (2011). Adiponectin receptor-1 expression is associated with good prognosis in gastric cancer. J Exp Clin Cancer Res.

[19] Hiyoshi M, Tsuno NH, Otani K, Kawai K, Nishikawa T, Shuno Y, Sasaki K, Hongo K, Kaneko M, Sunami E, Takahashi K, Nagawa H, Kitayama J (2012). Adiponectin receptor 2 is negatively associated with lymph node metastasis of colorectal cancer. Oncol Lett.

[20] Byeon JS, Jeong JY, Kim MJ, Lee SM, Nam WH, Myung SJ, Kim JG, Yang SK, Kim JH, Suh DJ (2010). Adiponectin and adiponectin receptor in relation to colorectal cancer progression. Int J Cancer.

[21] Jeong YJ, Bong JG, Park SH, Choi JH, Oh HK (2011). Expression of leptin, leptin receptor, adiponectin, and adiponectin receptor in ductal carcinoma in situ and invasive breast cancer. J Breast Cancer.

[22] Doyle SL, Donohoe CL, Finn SP, Howard JM, Lithander FE, Reynolds JV, Pidgeon GP, Lysaght J (2012). IGF-1 and its receptor in esophageal cancer: association with adenocarcinoma and visceral obesity. Am J Gastroenterol.

[23] Hoda MR, Keely SJ, Bertelsen LS, Junger WG, Dharmasena D, Barrett KE (2007). Leptin acts as a mitogenic and antiapoptotic factor for colonic cancer cells. Br J Surg.

[24] Vona-Davis L, and Rose DP (2009). Angiogenesis, adipokines and breast cancer. Cytokine Growth Factor Rev.

[25] Jung JI, Cho HJ, Jung YJ, Kwon SH, Her S, Choi SS, Shin SH, Lee KW, Park JH (2015). High-fat diet-induced obesity increases lymphangiogenesis and lymph node metastasis in the B16F10 melanoma allograft model: Roles of adipocytes and M2-macrophages. Int J Cancer.

[26] Clasper S, Royston D, Baban D, Cao Y, Ewers S, Butz S, Vestweber D, Jackson DG (2008). A novel gene expression profile in lymphatics associated with tumor growth and nodal metastasis. Cancer Res.

[27] Silha JV, Krsek M, Sucharda P, Murphy LJ (2005). Angiogenic factors are elevated in overweight and obese individuals. Int J Obes (Lond).

[28] Hausman GJ, and Richardson RL (2004). Adipose tissue angiogenesis. J Anim Sci.

[29] Guo S, Liu M, Wang G, Torroella-Kouri M, Gonzalez-Perez RR (2012). Oncogenic role and therapeutic target of leptin signaling in breast cancer and cancer stem cells. Biochim Biophys Acta.

[30] Yan D, Avtanski D, Saxena NK, Sharma D (2012). Leptin-induced epithelial-mesenchymal transition in breast cancer cells requires beta-catenin activation via Akt/GSK3- and MTA1/Wnt1 protein-dependent pathways. J Biol Chem.

[31] Feng H, Liu Q, Zhang N, Zheng L, Sang M, Feng J, Zhang J, Wu X, Shan B (2014). Leptin promotes metastasis by inducing an epithelial-mesenchymal transition in A549 lung cancer cells. Oncol Res.

[32] Zhao L, Shen ZX, Luo HS, Shen L (2005). Possible involvement of leptin and leptin receptor in developing gastric adenocarcinoma. World J Gastroenterol.

[33] Koda M, Sulkowska M, Kanczuga-Koda L, Surmacz E, Sulkowski S (2007). Overexpression of the obesity hormone leptin in human colorectal cancer. J Clin Pathol.

[34] Howard JM, Beddy P, Ennis D, Keogan M, Pidgeon GP, Reynolds JV (2010). Associations between leptin and adiponectin receptor upregulation, visceral obesity and tumour stage in oesophageal and junctional adenocarcinoma. Br J Surg.

[35] Shah NR, and Braverman ER (2012). Measuring adiposity in patients: the utility of body mass index (BMI), percent body fat, and leptin. PLoS One.

[36] Ando S, and Catalano S (2012). The multifactorial role of leptin in driving the breast cancer microenvironment. Nat Rev Endocrinol.

[37] Howard JM, Pidgeon GP, Reynolds JV (2010). Leptin and gastro-intestinal malignancies. Obes Rev.

[38] Riondino S, Roselli M, Palmirotta R, Della-Morte D, Ferroni P, Guadagni F (2014). Obesity and colorectal cancer: role of adipokines in tumor initiation and progression. World J Gastroenterol.

[39] Klein S, Allison DB, Heymsfield SB, Kelley DE, Leibel RL, Nonas C, Kahn R (2007). Waist circumference and cardiometabolic risk: a consensus statement from Shaping America's Health: Association for Weight Management and Obesity Prevention; NAASO, The Obesity Society; the American Society for Nutrition; and the American Diabetes Association. Am J Clin Nutr.

[40] Neeland IJ, Ayers CR, Rohatgi AK, Turer AT, Berry JD, Das SR, Vega GL, Khera A, McGuire DK, Grundy SM, de Lemos JA (2013). Associations of visceral and abdominal subcutaneous adipose tissue with markers of cardiac and metabolic risk in obese adults. Obesity (Silver Spring).

[41] Bain GH, Collie-Duguid E, Murray GI, Gilbert FJ, Denison A, McKiddie F, Ahearn T, Fleming I, Leeds J, Phull P, Park K, Nanthakumaran S, Grabsch HI, Tan P, Welch A, Schweiger L, Dahle-Smith A, Urquhart G, Finegan M, Petty RD (2014). Tumour expression of leptin is associated with chemotherapy resistance and therapy-independent prognosis in gastro-oesophageal adenocarcinomas. Br J Cancer.

[42] Park J, Euhus DM, Scherer PE (2011). Paracrine and endocrine effects of adipose tissue on cancer development and progression. Endocr Rev.

[43] Scarpace PJ, Matheny M, Strehler KY, Toklu HZ, Kirichenko N, Carter CS, Morgan D, Tumer N (2015). Rapamycin Normalizes Serum Leptin by Alleviating Obesity and Reducing Leptin Synthesis in Aged Rats. J Gerontol A Biol Sci Med Sci.

[44] Leontieva OV, Paszkiewicz GM, Blagosklonny MV (2014). Comparison of rapamycin schedules in mice on high-fat diet. Cell Cycle.

[45] Blagosklonny MV (2012). Common drugs and treatments for cancer and age-related diseases: revitalizing answers to NCI's provocative questions. Oncotarget.

[46] Edge SB, and Compton CC (2010). The American Joint Committee on Cancer: the 7th edition of the AJCC cancer staging manual and the future of TNM. Ann Surg Oncol.

[47] Ancona E, Ruol A, Santi S, Merigliano S, Sileni VC, Koussis H, Zaninotto G, Bonavina L, Peracchia A (2001). Only pathologic complete response to neoadjuvant chemotherapy improves significantly the long term survival of patients with resectable esophageal squamous cell carcinoma: final report of a randomized, controlled trial of preoperative chemotherapy versus surgery alone. Cancer.

[48] Ruol A, Castoro C, Portale G, Cavallin F, Sileni VC, Cagol M, Alfieri R, Corti L, Boso C, Zaninotto G, Peracchia A, Ancona E (2009). Trends in management and prognosis for esophageal cancer surgery: twenty-five years of experience at a single institution. Arch Surg.

