# Supplementary material for: Esophageal adenocarcinoma and obesity: peritumoral adipose tissue plays a role in lymph node invasion
Source: Oncotarget. 2015 Mar 14;6(13):11203–15. doi: 10.18632/oncotarget.3587 (PMC4484450; doi:10.18632/oncotarget.3587)
Supplement: Supplementary file 1 [file oncotarget-06-11203-s001.pdf]

Esophageal adenocarcinoma and obesity: peritumoral adipose tissue plays a role in lymph node invasion

Supplementary Material

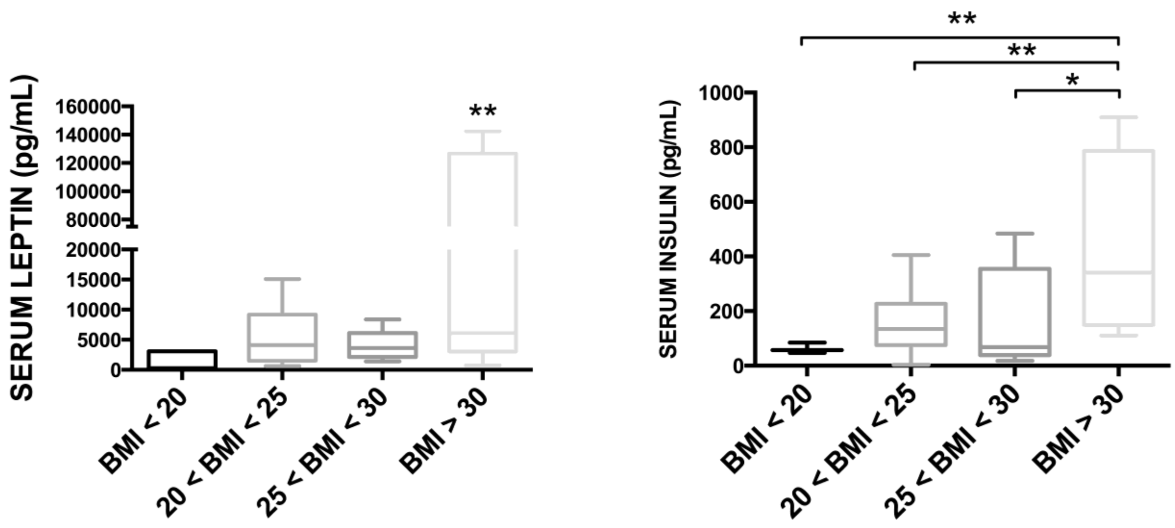

Supplementary figure 1: Body Mass Index (BMI) and obesity-related parameters in ADK patients. BMI of patients was retrieved at the time of surgery. BMI values were directly correlated with leptin and insulin levels in the serum of EAC patients. *$p < 0.05$ and **$p < 0.01$

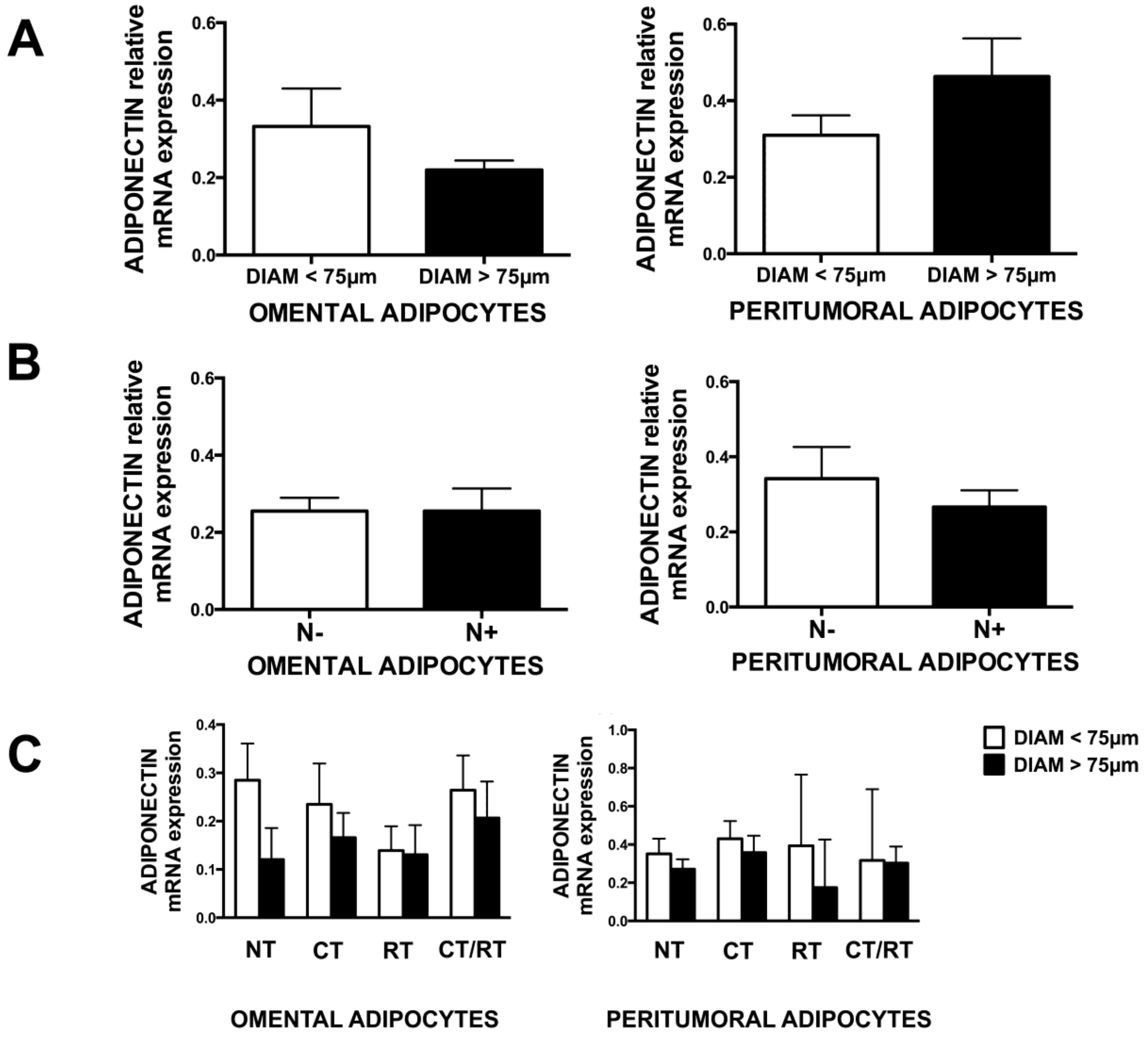

Supplementary figure 2: Adiponectin expression and adipocyte size in omental and peritumoral adipose tissue. Total RNA was isolated from visceral adipose tissue samples of patients with EAC. Adipocyte diameters were measured in hematoxylin and eosin stained sections of the same samples. (A) Adiponectin mRNA expression was measured using qRT-PCR with HMBS as internal control. (B) Adipocyte size and adiponectin expression were measured in lymph node metastasis negative (N-) or positive (N+) EAC patients. (C) EAC patients were divided into groups based on their treatment with chemotherapy (CT), radiotherapy (RT), combined chemotherapy and radiotherapy (CT/RT) or no treatment (NT). Non-parametric statistical tests were used.

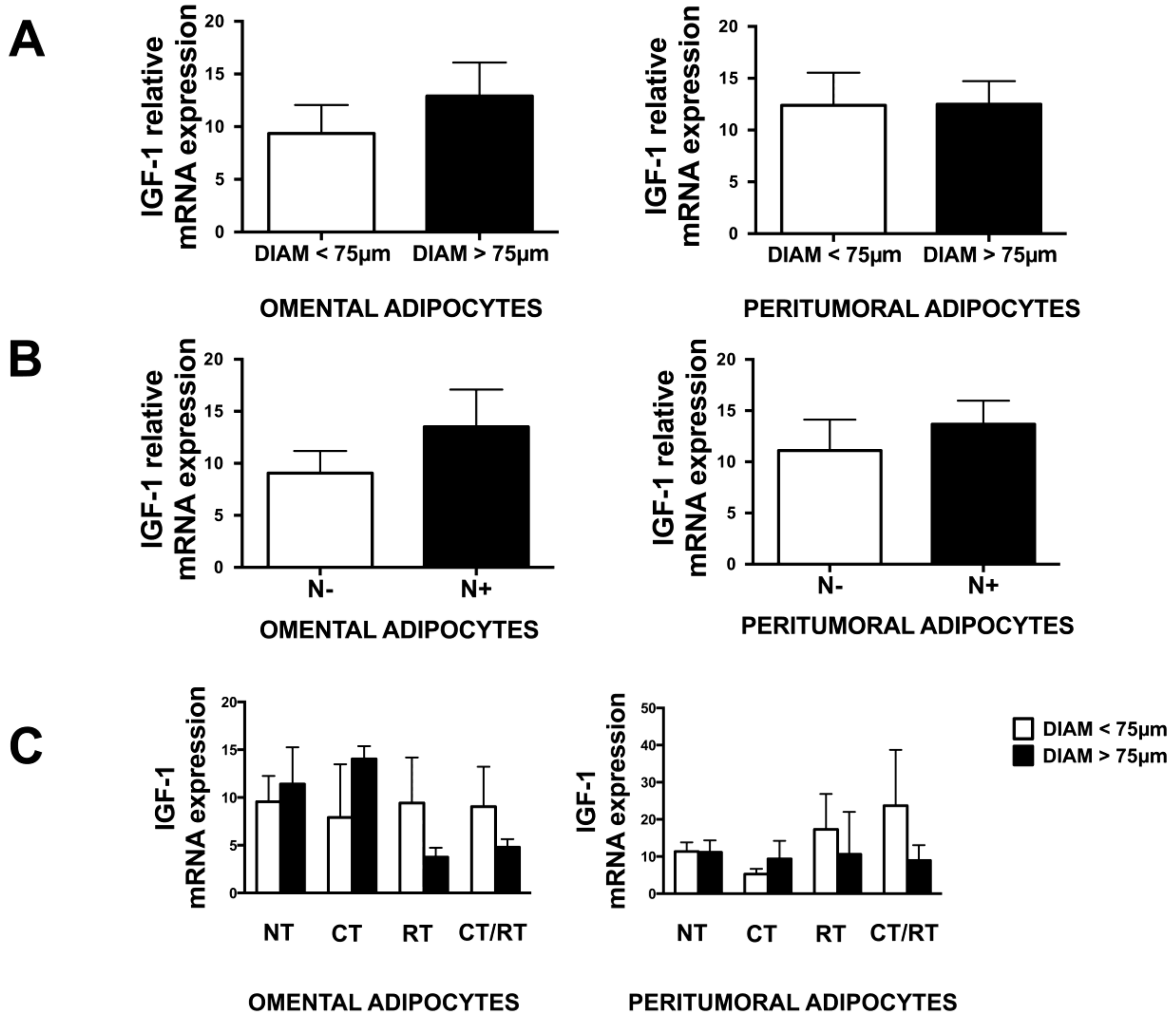

Supplementary figure 3: IGF-1 expression and adipocyte size in omental and peritumoral adipose tissue. Total RNA was isolated from visceral adipose tissue samples of patients with EAC. Adipocytes diameters were measured in hematoxylin and eosin stained sections of the same samples. (A) IGF-1 mRNA expression was measured using qRT-PCR with HMBS as internal control. (B) Adipocyte size and IGF-1 expression were measured in lymph node metastasis negative (N-) or positive (N+) EAC patients. (C) EAC patients were divided into groups based on their treatment with chemotherapy (CT), radiotherapy (RT), combined chemotherapy and radiotherapy (CT/RT) or no treatment (NT). Non-parametric statistical tests were used.

Supplementary table 1: Real-Time PCR primer sequences. Specific intron-spanning primers were designed to measure mRNA levels of human genes by using Real-Time PCR.

**Human gene**	**Forward sequence**	**Reverse sequence**
**HMBS**	5'-GGCAATGCGGGCTGCAA-3'	5'-GGGTACCCACGCGAATCAC-3'
**Leptin**	5'-GTGCGGATTCTTGTGGCTTT-3'	5'-GGAATGAAGTCCAAACCGGTG-3'
**Adiponectin**	5'-CAATCCCACCACTGAATTGCTG-3'	5'-CCTGGTGAGAAGGGTGAGAA-3'
**ObR**	5'-TGTTGGGAAGTTGGCACATTG-3'	5'-TGGGAAGATGTTCCGAACCCC-3'
**ADIPOR1**	5'-CGGCGGGGAGTTTAGAAGACCAAAC-3'	5'-TGCAGCTTCAGCTTGGGGAAAGG-3'
**α-SMA**	5'-AAGGCCAACCGGGAGAAAAT-3'	5'-ATTGTGGGTGACACCATCTCC A-3'
**E-Cadherin**	5'-ACGAGGCTAACGTCGTAATCA-3'	5'-TACTGCTGCTTGGCCTCAAA-3'
**IGF-1**	5'-GATGCACACACCATGTCCTCC -3'	5'-GCCTCCTTAGATCAGAGCTCC -3'
